# Probing Biological Nitrogen Fixation in Legumes Using Raman Spectroscopy

**DOI:** 10.3390/s24154944

**Published:** 2024-07-30

**Authors:** Abdolabbas Jafari, Kritarth Seth, Armin Werner, Shengjing Shi, Rainer Hofmann, Valerio Hoyos-Villegas

**Affiliations:** 1 Lincoln Agritech, Lincoln University, Lincoln 7647, New Zealand; wernera@lincolnagritech.co.nz; 2 AgResearch, Lincoln 7608, New Zealand; kritarth.seth@agresearch.co.nz (K.S.); shengjing.shi@agresearch.co.nz (S.S.); 3 Plant Biology Department, Lincoln University, Lincoln 7647, New Zealand; rainer.hofmann@lincoln.ac.nz; 4 Faculty of Agricultural and Environmental Sciences, McGill University, Montreal, QC H3A 0G4, Canada; valerio.hoyos-villegas@mcgill.ca

**Keywords:** pattern recognition, chemometrics, biosystems, precision agriculture, non-destructive, rhizobia, symbiosis

## Abstract

Biological nitrogen fixation (BNF) by symbiotic bacteria plays a vital role in sustainable agriculture. However, current quantification methods are often expensive and impractical. This study explores the potential of Raman spectroscopy, a non-invasive technique, for rapid assessment of BNF activity in soybeans. Raman spectra were obtained from soybean plants grown with and without rhizobia bacteria to identify spectral signatures associated with BNF. δN^15^ isotope ratio mass spectrometry (IRMS) was used to determine actual BNF percentages. Partial least squares regression (PLSR) was employed to develop a model for BNF quantification based on Raman spectra. The model explained 80% of the variation in BNF activity. To enhance the model’s specificity for BNF detection regardless of nitrogen availability, a subsequent elastic net (Enet) regularisation strategy was implemented. This approach provided insights into key wavenumbers and biochemicals associated with BNF in soybeans.

## 1. Introduction

Biological nitrogen fixation (BNF) is a critical process in sustainable agriculture. It provides a cost-effective alternative to synthetic nitrogen fertilisers, reducing reliance on non-renewable resources and potential environmental impacts [1,2]. However, accurately measuring BNF activity remains a challenge. Existing methods, such as the acetylene reduction assay, natural abundance method, and δN^15^ isotope dilution method, all have limitations. The natural abundance method, while considered the most accurate, is destructive and time-consuming [2]. Isotope ratio mass spectrometry (IRMS) offers a direct measure of N_2_ fixation using ^15^N_2_ incorporation [2,3], but it is not suitable for high-throughput screening or field applications due to its complexity and cost. Nitrogen balance calculations, based on long-term changes in soil and crop nitrogen content, can estimate potential BNF but lack the precision of direct measurement [2].

In recent years, Raman spectroscopy (RS) has emerged as a promising tool in plant biology and agriculture [4]. This non-destructive technique analyses the vibrational modes of molecules within a sample, generating a characteristic “fingerprint” spectrum [4]. Compared with traditional methods, RS offers several advantages: it is non-destructive, requires minimal sample preparation, and allows simultaneous analysis of multiple metabolites [2,5,6].

Legumes, through symbiosis with bacteria in their root systems, fix atmospheric nitrogen (N_2_) and convert it into usable forms. This process leads to the production of amino acids, which are primarily synthesised in the roots and transported to the aerial parts of the plant for essential processes like photosynthesis. RS has been successfully applied to analyse various plant metabolites, including amino acids [7], sugars [8,9], and nucleic acids [10]. Additionally, it has been used to study secondary metabolites in medicinal plants, detect plant stress responses, and analyse plant growth regulators [11]. Notably, RS has also been used to analyse cultured bacteria extracted from the rhizosphere, enabling the investigation of plant-microbe interactions [12]. Furthermore, studies have demonstrated the potential of RS gas analysis within closed chambers for the continuous monitoring of plant gas exchange, including N_2_ fixation rates [13]. However, macro-scale Raman measurements of plant organs can be challenging due to variations in chemical composition across different tissues. Leaves, stems, and roots have distinct chemical profiles, and even within the same organ, variations can occur depending on location (e.g., distance from the petiole) [14]. Additionally, the orientation of molecules within the tissue can influence the polarised Raman spectra [14]. These variations necessitate targeted measurements from specific regions of the plant or the use of chemometric techniques to account for and analyse such differences.

This study explores the potential of RS as a non-invasive, high-throughput method for measuring BNF activity in soybean plants. We hypothesise that BNF, by altering the plant’s metabolic state and potentially affecting energy consumption, could either directly influence the Raman spectra of plant tissues or indirectly lead to changes in the composition of other chemical compounds, ultimately resulting in spectral variations. We aimed to investigate whether these spectral changes can be linked to BNF activity, allowing for its quantification. Specifically, we sought to determine if differences in soybean plants’ nitrogen fixation levels could be distinguished through variations in their leaf Raman spectra.

## 2. Materials and Methods

Previous studies have established that nitrogen concentration in the growth medium reduces nitrogen fixation in legumes [15,16,17,18]. Therefore, in this study, nitrogen concentration was used as an agent to provide different levels of nitrogen fixation. Nitrogen fixation is suppressed when there is adequate nitrogen in the rooting medium, as the exchange of nitrogen for carbohydrates between plants and bacteria is halted. This means that legumes no longer provide their carbohydrates to host rhizobia in return for fixed nitrogen, which comes at an energy cost to the plant [19]. Using this phenomenon, we prepared growth media with varying nitrogen concentrations to achieve different nitrogen fixation rates. Specifically, we grew soybean plants in vermiculite using four different nitrogen concentrations (2 mM, 6 mM, 10 mM, and 14 mM solutions of ammonium nitrate) under controlled conditions in a growth chamber, with five replications for each treatment.

Soybean plants (*Glycine max* L. cv. Sonya, non-genetically modified) were grown in a closed, semi-sterile system for six weeks. To ensure precise control over nitrogen availability, the plants were cultivated in 4-litre pails (*n* = 56) filled with vermiculite (grade 2; compressed). Each box was filled with 2.2 L McKnights solution [20,21] and autoclaved (30 min, wet cycle, 121 °C) to eliminate any background rhizobia present. Before autoclaving, the lids of the boxes were drilled to make a 1.5 cm hole in the center for the plant stem to emerge. Soybean seeds were surface sterilised (70% ethanol, 30 s; 1% NaOCl, 2 min; 4X rinses, sterilised deionised water, 30 s) and pre-germinated for 6 days (22 °C; dark) in a 5 cm deep bed of vermiculite and water (3:1; autoclaved) in trays.

Healthy seedlings were then transplanted into the boxes under sterile conditions, carefully placed through the holes on the lids, at one seedling per box. The seedlings were allowed to establish for 2 days in a clean growth room with 600 µmol/m^2^/s of light for 16 h day at 24 °C and 8 h night at 19 °C. Rhizobia-inoculated plants were inoculated with a pipette at the base of the seedling with 1 × 10^7^ colony forming units (cfu) of *Bradyrhizobium japonicum* Strain ICMP3163 (Landcare research, Lincoln, New Zealand) twice, on day 1 (day of inoculation; 8 days from germination) and again on day 8 to give the seedlings the maximum chance of nodule formation. Nitrogen (N) was added as 5% atom ^15^N ammonium nitrate (Sigma Aldrich, St. Louis, MO, USA) at concentrations of 2, 6, 10, and 14 mM of N. To account for increasing N requirements of the plant while growing, N was not added all at once, but was split into four doses of 10%, 20%, 30%, and 40% added on days 1, 8, 15, and 22, respectively. Plants were watered with sterilised deionised water as and when needed.

To establish a reliable model, we employed isotope ratio mass spectrometry (IRMS) to measure BNF rates, which will serve as the ground truth for subsequent analyses.

### 2.1. Experimental Design

Achieving varying levels of nitrogen fixation (BNF) for treatment design presented a challenge. Ideally, we sought a manipulation method that would not directly alter plant chemistry, as this could confound spectral data analysis. However, we utilised nitrogen availability as a tool to indirectly control BNF. While this approach influences spectral features, it becomes difficult to disentangle whether observed spectral variations primarily reflect changes in BNF activity or simply the introduced nitrogen (ammonium nitrate) itself.

**Figure 1:**
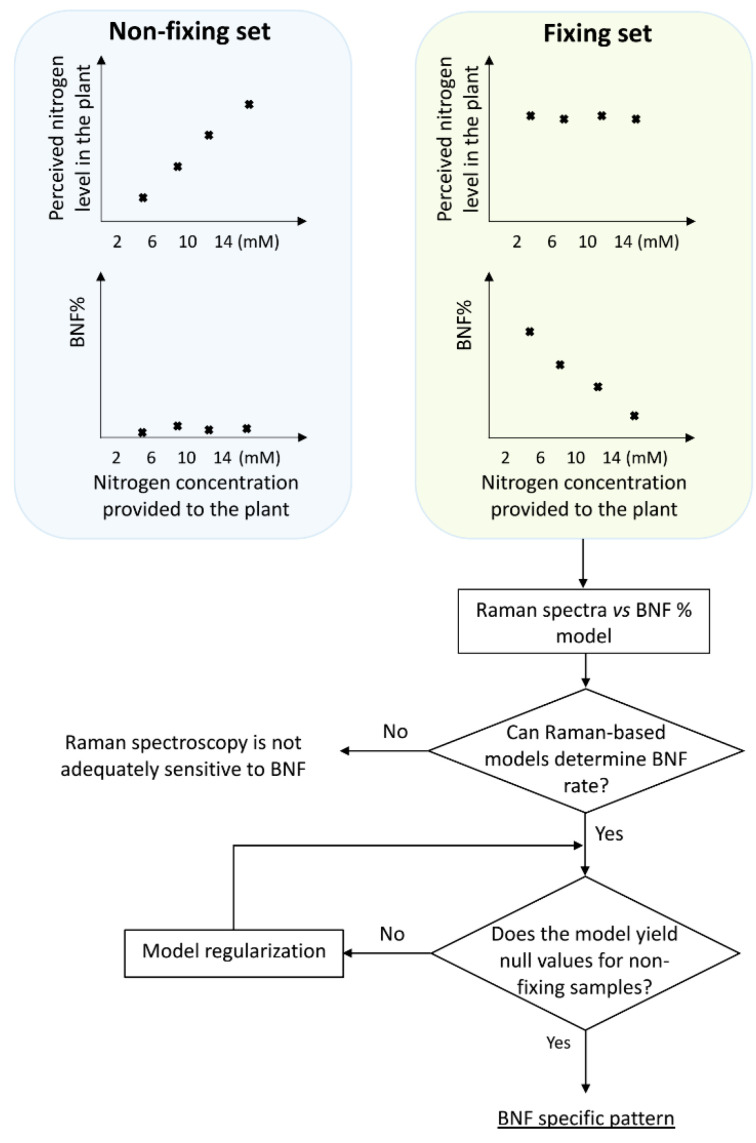
Experimental design and procedure for extracting BNF specific pattern from Raman spectra of the aerial parts of soybean plants. Spectra were collected at six weeks after inoculation, when the total nitrogen content became consistent across all treatments within the nitrogen-fixing set.

To distinguish between spectral features arising from BNF activity and those solely due to nitrogen variation, we employed two treatment sets: fixing and non-fixing (Figure 1). Soybean samples in the non-fixing set were grown under completely sterile conditions to ensure that no bacteria for nitrogen fixation existed in the growing medium, while the other set was inoculated with *Bradyrhizobium japonicum* bacteria.

We initiated the procedure by developing a model using the Raman data from the fixing set, enabling it to take Raman spectra of the leaves as input and provide nitrogen fixation rates as output. To achieve this, the input data were needed to cover the full range of nitrogen fixation variations. In addition, it is important to note that while we provided the plants with varying nitrogen concentrations, this did not guarantee a uniform distribution of BNF rates across the range. To address this, we conducted δN^15^ measurements to assess BNF variations in the samples and determine the nitrogen accumulated in the plants at the time of measurement.

The schematic illustration in Figure 1 explains the experimental design, including the division of plants into two groups: those inoculated with nitrogen-fixing bacteria (BNF set) and those grown without bacteria (non-fixing set). The figure also depicts the expected observations of the two groups under varying nitrogen treatments. This design acts as a key control experiment to validate the specificity of the models in BNF measurements. We compared the Raman spectral patterns of both groups to assess if the model captures general nitrogen responses or changes specific to nitrogen fixation. If the spectral patterns in both sets followed the same trends with varying nitrogen levels, it would indicate the model might be capturing general nitrogen responses rather than changes specific to nitrogen fixation. This would suggest the method is unsuitable for BNF quantification.

Our δN^15^ isotope ratio mass spectrometry (IRMS) experiments confirmed that at six weeks after germination, soybean plants in the BNF set achieved a mature symbiotic state with the bacteria fully supplying their nitrogen needs. Therefore, it is reasonable to expect that six weeks after inoculation, there should be no discernible differences in N-related traits within the spectra of fixing plants, while the N-related distinctions may persist in the non-fixing set. Nevertheless, it is worth exploring the possibility of residual effects from prior nitrogen deficiency in the spectra of fixing plants. For a robust method specifically measuring BNF (independent of nitrogen variations), evaluation with non-fixing samples is crucial. This ensures the model does not simply detect general nitrogen variation.

### 2.2. Raman Spectra Acquisition

Raman spectra were collected from the first fully developed leaves of soybean plants using a Horiba iHR320 spectrometer (Horiba, Kyoto, Japan). To optimise signal quality while minimising plant tissue fluorescence, a 785 nm laser source with 350 mW power was utilised for excitation.

The leaves were placed on the acquisition stage of the spectrometer, and the laser was focused on the leaf surface without any sample preparation. To ensure precise positioning of all sampling spots within the focal point of the laser beam, a fixture was used to securely hold the leaves, preventing deformation and local undulation of the leaf surface.

Raman spectra were collected from the samples across the range of 400 cm^−1^ to 1800 cm^−1^. Each spectrum was acquired with a 20-s acquisition time and three accumulations. For improved signal-to-noise ratio (S/N), three measurements were taken from nearly the same spot on the leaves. To mitigate potential dehydration or burning of the leaf texture due to the laser intensity and acquisition time, the data acquisition spot was slightly shifted by approximately ±2 mm in each iteration relative to the initial spot.

To validate the accuracy of our Raman detection in capturing the chemical composition related to nitrogen fixation, we conducted a comparative analysis using known plant chemicals, including allantoin, β-carotene, pectin, and glycerol. By comparing the experimentally obtained Raman spectra of these compounds with established spectral databases, we were able to confirm the reliability of our Raman system in identifying key functional groups associated with these molecules.

### 2.3. Data Processing and Analysis

The collected spectra underwent data preprocessing steps, which involved removing cosmic rays and smoothing to eliminate random noise. Various combinations of polynomial orders and kernel sizes were tested using Savitzky–Golay filters. Ultimately, a filter with a polynomial order of 13 and a frame length of 51 demonstrated optimal performance by effectively eliminating random noise while preserving essential Raman peaks. As a result, this specific filter configuration was chosen for subsequent processing steps.

For excitation, a 785 nm laser source was chosen. Shorter wavelengths, like 532 nm, can generate a significantly higher level of fluorescence, especially in some biological materials such as plant leaves. This fluorescence can overwhelm the weaker Raman signal, making it difficult to analyse the desired Raman spectrum. Conversely, longer wavelengths, such as 1064 nm, while minimising fluorescence, often suffer from a significantly weaker Raman scattering effect [22]. Therefore, 785 nm was used as a compromise between minimising fluorescence and maximising the Raman signal intensity for our samples.

Even with the utilisation of a 785 nm laser, the Raman spectra of the soybean samples still showed significant fluorescence intensity. To address this, a baseline detection procedure [23] was employed, with a modification that used an iterative moving average approach instead of polynomial kernels. The modification was justified based on a comparison between the results obtained from the two types of kernels, i.e., moving average versus polynomials. The iterative moving average approach was found to be more effective in reducing fluorescence without distorting the underlying Raman signal.

Initial evaluation of baseline-subtracted spectra revealed that a kernel size of 50 wavenumbers with 50 iterations yielded optimal performance in isolating pure Raman peaks from the fluorescent background using a MATLAB implementation.

To detect BNF-related patterns, partial least squares regression (PLSR) models were developed using Raman data from the leaves of “Fixing set” plants. PLSR is a statistical technique that relates a set of predictor variables (X) to a set of response variables (Y) by extracting latent variables (underlying factors) that explain the maximum covariance (shared variance) between X and Y. Unlike standard regression, which directly models Y based on X, PLSR finds a set of latent variables that capture the most important information in both X and Y. It then uses these latent variables to build a model that predicts Y. It is mostly useful when there are many correlated predictor variables (multicollinearity) which can cause problems in standard regression [24].

The calibration set for developing the model comprised soybean samples from the nitrogen-fixing set. Model performance was assessed using the coefficient of determination (R^2^) and root mean square error (RMSE) for both calibration and cross-validation sets. Leave-one-out cross-validation (LOOCV) was employed, where each soybean sample from the nitrogen-fixing set was sequentially excluded from the training set and used as a validation set. This iterative process provided a robust estimate of model performance.

### 2.4. Model Regularisation

The model’s target BNF estimation for the non-fixing samples was expected to be zero. To refine the BNF information from the Raman spectra of the N-related variations due to nitrogen concentrations applied to the root medium, model regularisation was needed. As illustrated in the final stage of Figure 1, this regularisation procedure was carried out using elastic net (ENet) regularisation.

This procedure involves adjusting the influential vectors of the model to achieve a target null value for the non-fixing set. While this adjustment may reduce the model’s performance on the fixing set, it results in a more specific BNF determination model. We determined the trade-off point between maximising BNF sensitivity and minimising N sensitivity by applying elastic net regularization.

While this study employs the concept of regularisation, it differs from the typical approach. Here, we do not adjust the model to reduce variance on the validation set. Instead, regularisation is used to force the model to predict the lowest possible values for the non-fixing set, minimising its sensitivity to nitrogen concentration. The resulting performance metrics will then indicate the extent of BNF-representative information captured within the Raman spectra.

Elastic net (EN) regularisation is a hybrid approach that combines L1 (lasso regularisation) and L2 (Tikhonov or ridge regularisation) methods. In all three techniques (L1, L2, and EN), regularisation terms, also known as penalties, are added to the ordinary least squares cost functions. These penalties aim to minimise the model’s variance.

The regularisation strength is typically controlled by the hyperparameter labmda (λ) which determines the amount of shrinkage applied to the coefficients. The model coefficients, β and β_0_ are calculated by minimising the cost function J(β) [25] as follows:

(1) $$J\left(\beta \right)=\frac{1}{2N}(\sum_{i=1}^{N}{{(y}_{i}-\beta_{0}-x_{i}^{T}\beta )}^{2}+\lambda P_{\alpha }(\beta )$$

where

(2) $$P_{\alpha }\left(\beta \right)=\frac{\left(1-\alpha \right)}{2}{\left\|\beta \right\|}_{2}^{2}+\alpha {\left\|\beta \right\|}_{1}=\sum_{j=1}^{P}\left(\frac{\left(1-\alpha \right)}{2}\beta_{j}^{2}+\alpha \left|\beta_{j}\right|\right)$$

P_α_(β): Elastic net’s penalty term

We aimed to reduce the sensitivity of the model to factors mostly indicative of nitrogen concentration, ideally achieving the lowest possible value for this plant set. The regularisation process will determine the optimal λ that yields the lowest BNF for the non-fixing plants. An equal balance of L1 and L2 penalties (α = 0.5) was incorporated into the cost function. While this regularisation might decrease model performance for the fixing set, it tailors the model specifically for BNF measurement.

## 3. Results and Discussion

The Raman spectra of both “Fixing” and “Non-fixing” soybean leaves exhibited broadly similar patterns, with the most prominent variations occurring at the peaks around 747 cm^−1^, 1160 cm^−1^, and 1525 cm^−1^. These peaks are associated with molecular vibrations of specific biomolecules including pectin [26] and carotenoids [7].

**Figure 2:**
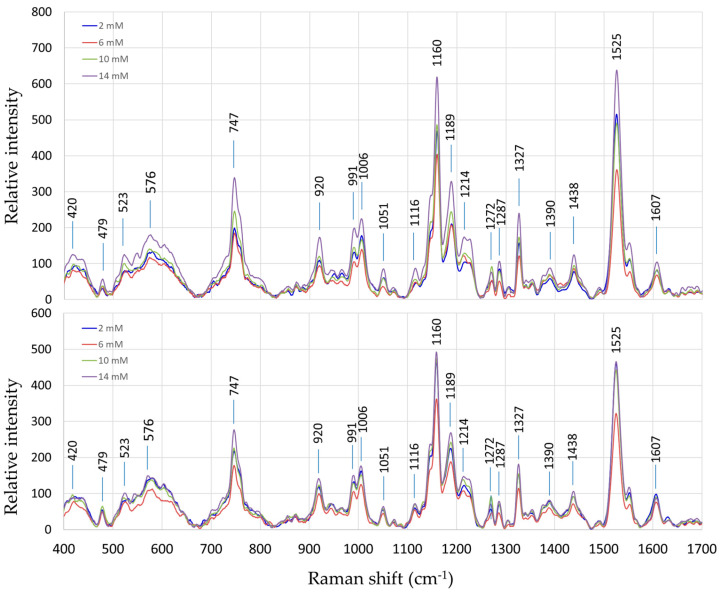
Average Raman spectra of soybean leaves under different nitrogen treatments; nitrogen-fixing samples (**top**) and non-fixing samples (**bottom**).

The average Raman spectra of soybean leaves under different nitrogen treatments are shown in Figure 2 for the two sets of soybean plant samples, i.e., nitrogen-fixing vs. non-fixing sets. Each spectrum represents an average of 15 measurements, including five replicates of three measurement points each.

A comparison between the spectra of the fixing and non-fixing sets reveals that an easily inferable trend in the variations of the Raman intensities is not readily discernible at any specific Raman shift points. This indicates that BNF-related metabolites did not explicitly change the Raman spectrum. Instead, the information on BNF activities is embedded within the Raman data and must be extracted using sophisticated chemometric techniques such as PLSR, as explained in the Methods section.

**Figure 3:**
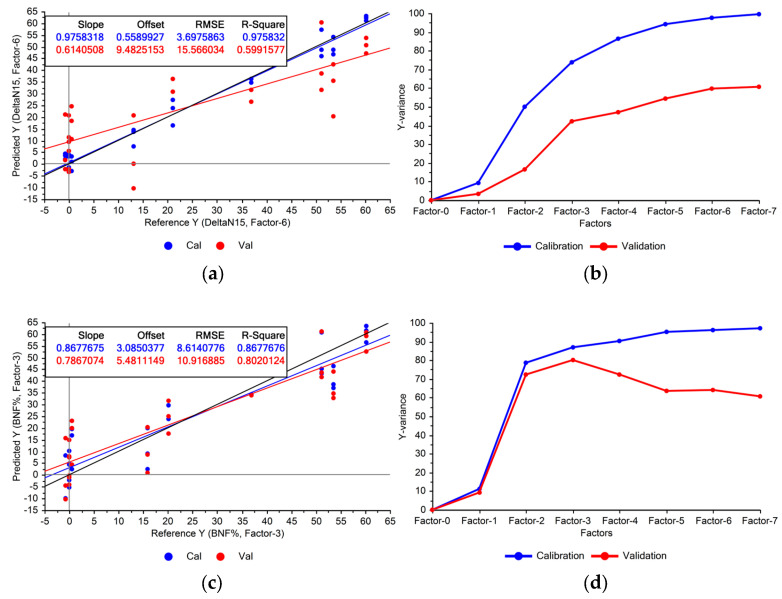
Performance of the BNF estimation models using Raman spectra acquired from fixing plants for calibration (Cal) and validation (Val) datasets: (**a**) using full Raman spectrum (600–1770 cm^−1^), (**c**) using selected Raman peaks. Explained variances by PLSR factors are shown in: (**b**) full Raman spectrum, (**d**) selected Raman peaks.

As illustrated in the first stage of Figure 1, the PLSR models were developed using the Raman data from the “Fixing set”. The performance of the models indicated the feasibility of estimating BNF from the leaves’ spectra (Figure 3a).

### 3.1. Model Optimisation

We evaluated various normalisation and standardisation techniques, including area normalisation, standard normal variate (SNV), and multiplicative scatter correction (MSC), on the model’s performance. This evaluation showed a reduction rather than an improvement in performance. Consequently, we concluded that the absolute intensity of the Raman data is as crucial as the relative intensity of the Raman shifts. Therefore, normalising or standardising the data would result in the loss of important information.

Figure 3a illustrates that the constructed model exhibited significant variance, indicating notable disparities between calibration and cross-validation accuracies. This suggests overfitting, where the model memorises training data variations rather than capturing underlying patterns, compromising its generalisability to unseen data. Addressing this issue may involve removing features with low importance from the model.

Using the entire Raman spectrum in the modelling process can lead to overfitting due to the numerous features involved, increasing the chance of memorisation. The divergence between the variances explained by the model during training and cross-validation (Figure 3b) is another sign of potential overfitting. While omitting certain features might reduce performance on the calibration set, it could improve validation results and enhance the model’s generalisability. Given the already high explained variances on the training set (100% with seven factors), there is ample headroom to reduce the variance in the validation set (Figure 3b).

As the first step towards reducing variables in the model, we used Raman peaks instead of the entire spectrum. Unlike NIR spectroscopy and hyperspectral imaging, Raman peaks directly correspond to specific molecular bonds, indicating specific molecules or compounds. Therefore, a model using selected Raman peaks offers clearer interpretability. We evaluated model performance using only the distinctive peaks listed in Table 1, reducing the input features from 1097 wavenumbers to a smaller set of specific ones.

We used variable importance in projection (VIP) scores to identify the most influential features. Features with a VIP score below 1 were excluded from the PLSR model. Figure 3c shows the model’s performance using only the selected Raman peaks for BNF estimation. The explained variances (Figure 3d) suggest improved generalisability compared with the model using the entire spectrum. The model achieved optimal performance with the first three PLS factors, capturing 80% of the variation within the selected Raman peaks. This aligns with expectations, as nitrogen fixation did not occur in the non-fixing set grown under sterile conditions, confirmed by the absence of root nodules.

These findings validate our initial assumption that specific features within the Raman spectra of leaves can reflect nitrogen fixation rates. To further confirm this, we evaluated the model’s performance using spectra from the non-fixing sets grown under different nitrogen treatments.

### 3.2. Regularisation Results

**Figure 4:**
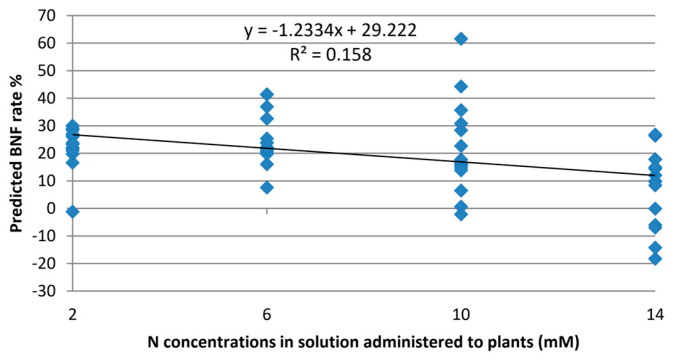
Output of the BNF model on Raman spectra collected from “non-fixing” soybean plants with various levels of nitrogen fixation.

To assess the model’s ability to estimate BNF exclusively, irrespective of nitrogen levels, it was validated using Raman spectra from leaves of the non-fixing sets. The model yielded an average value of 13.7% BNF for these plants. While the experiment aimed to eliminate the influence of nitrogen variations, Figure 4 reveals some, albeit minor, correlations between estimated BNF and nitrogen treatment levels. This suggests that some features within the Raman spectra of the leaves still capture information about nitrogen variations in the plant.

To further refine the BNF-related Raman peaks and potentially identify the associated chemical compounds, regularisation was employed. The aim of regularisation in this study was to decrease the sensitivity of the model to features (wavenumbers) associated with nitrogen concentration rather than nitrogen fixation.

**Figure 5:**
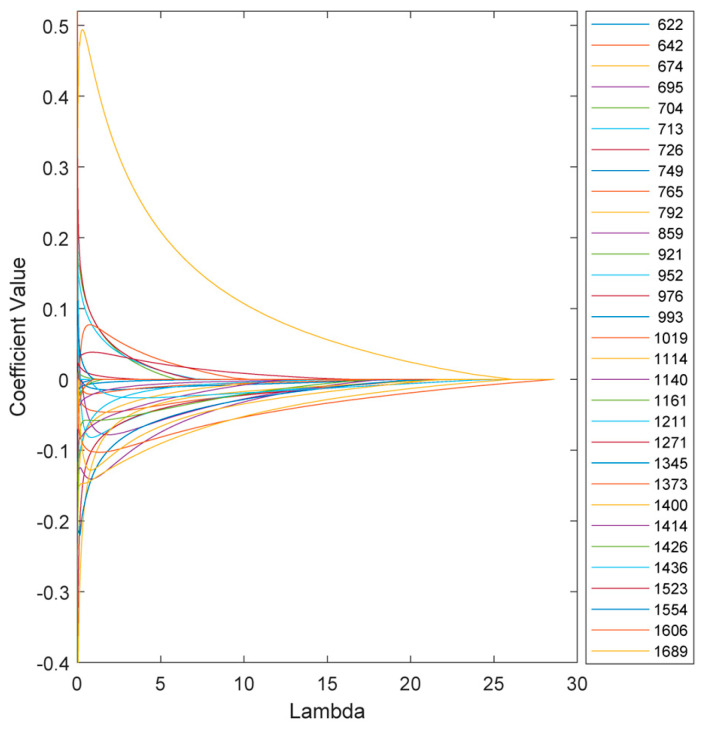
Variation in the ENet model coefficients at various λ values.

The variation in the model coefficients at various λ values is depicted in Figure 5. Increasing λ increases the penalties on the model, which in turn reduces sensitivity and, in some cases, eliminates certain features depending on the chosen threshold.

**Figure 6:**
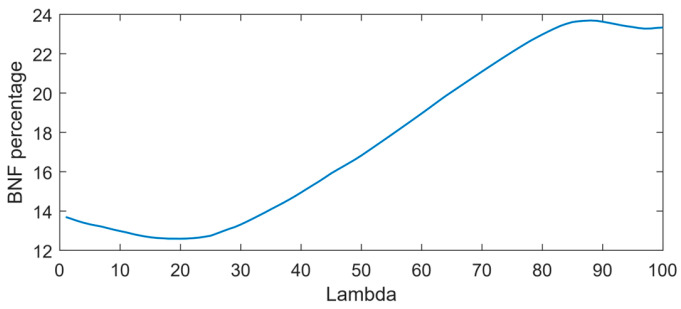
Output of the ENet model for non-fixing soybean samples at various λ values.

The optimal combination of coefficients was determined based on the minimum output of the elastic net (ENet) model at various λ values. Figure 6 depicts the optimal point at λ = 20, where the non-fixing set achieves a minimum BNF output of 12.6.

**Figure 7:**
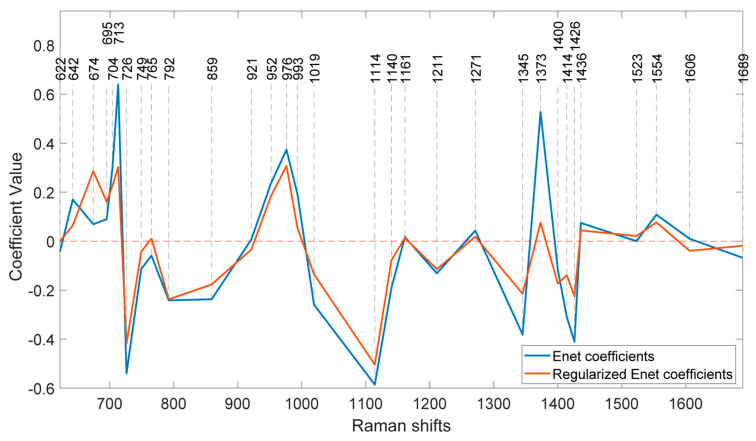
Coefficients of the ENet and regularised ENet models.

At this stage, the coefficients of the features in the model at the optimum λ can be determined. Figure 7 illustrates the coefficients of the model without regularisation (λ = 0) and at the optimal regularisation (λ = 20) which corresponds to a BNF% of 12.6% for the non-fixing set.

Once the optimal λ value is determined, the coefficients associated with each feature in the model can be analysed. Figure 7 compares the model coefficients without regularisation (λ = 0) to those obtained with optimal regularisation (λ = 20). The latter achieves a minimum BNF value of 12.6% for the non-fixing set.

It is evident that the effects of regularisation were not uniform across all features. Wavenumbers with significantly reduced coefficient values after regularisation likely correspond to nitrogen concentration, suggesting a weaker contribution to biological nitrogen fixation (BNF). Additionally, the direction of change in coefficients varies across peaks. For example, the regularised model shows a higher coefficient at 674 cm^−1^ compared with the non-regularised model, while the coefficient at 713 cm^−1^ has decreased. Notably, some coefficients were eliminated entirely due to the L1 (lasso) penalty.

To identify the most BNF-related peaks, we can select wavenumbers that meet two criteria: (1) a coefficient greater than 0.1 in the regularised model and (2) a reduction of more than 10% in the coefficient value compared with the non-regularised model. Applying these criteria to the 31 features in Table 1 results in the selection of 15 key wavenumbers.

Interestingly, the two prominent bands at 1160 cm^−1^ and 1525 cm^−1^, previously associated with carotenoids in Figure 1, do not appear to play significant roles in biological nitrogen fixation (BNF) despite being strong indicators of plant greenness and nitrogen uptake.

Carotenoids typically exhibit two strong Raman bands due to polyene chain stretching vibrations [27]. As changes in nitrogen concentration directly influence leaf chlorophyll content, these peaks were expected to be prominent features in the nitrogen estimation models. However, Table 1 reveals that the key BNF-related peaks are not necessarily the strongest or most distinctive peaks in the leaf spectrum. Instead, the information on BNF appears to be encoded in Raman peaks with relatively lower intensity.

An interesting observation is that while the regularised model’s coefficients generally decrease across most peaks, the coefficient at 674 cm^−1^ actually increases. This peak is associated with C–C–O rocking and deformation vibrations in glycerol [28]. This finding is particularly intriguing given the high concentration of glycerol in soybeans [29]. It suggests a potential link between biological nitrogen fixation and glycerol content, warranting further investigation.

**Table 1:** Assignments of vibrational bands to the influential Raman peaks in BNF specific model developed for soybean plants.

Band (cm^−1^)	Vibrational Mode	Assignment
674	ν(C–C–O) + δ(C–C–O)	Carbohydrates [30], Glycerol [28]
695	δ(C–C–H) twisting	Cellulose Avicel (II) [31]
704	ring deformation	Cytosine [32]
713	C–S–C asymmetric stretch; H_2_O rocking	Isoleucine, Methionine [33]
726	ring breath	Adenine [34]
859	τ (C–N)	Arginine, Serine [33]
952	δ(CCH), δ(COH)	Pectin [26]
976	CH_2_ rocking	Glycerol [28]
1019	C–O–H deformation	Alcoholic hydroxyl [33]
1114	νs(C–O–C)	Glycosidic ring breathing [35], Cellulose [36]
1211	δ(>C=C–C–) β-ring	β-Carotene [37,38,39]
1345	ν(C–O); δ(C–O–H)	Carbohydrates [30]
1400	ν; δ(C–OH)COOH; δs(CH_3_)	Pectin [26], β-carotene [37], Cellulose [36]
1414	δ(C–H)	Deoxyadenosine triphosphate [28]
1426	νs(COO–); CH_2_ deformation	Aspartic acid, Glutamic acid, Methionine [28,33]

Soybeans are considered as a complete protein source, containing all nine essential amino acids. The peak at 713 cm^−1^ can be attributed to protein elements and amino acids such as isoleucine and methionine [33]. Isoleucine is essential for nitrogen metabolism, and changes in the source of nitrogen uptake may influence its production. The quantity of isoleucine in plant leaves varies depending on factors such as plant species, growth conditions, and leaf part. While plant leaves are generally not rich sources of isoleucine compared with animal products, soybeans stand out for their good isoleucine content [40]. The peak at 726 cm^−1^ can also be assigned to adenine [34], a fundamental building block of DNA and RNA. Peak positions can shift depending on factors like the pH of the environment. This peak is often attributed to in-plane ring breathing vibrations within the adenine molecule.

Serine and arginine are other essential amino acids for legumes, playing crucial roles in their growth and development, and they exhibit a prominent peak at 859 cm^−1^ [33]. A recent study [41] suggests that arginine serves as a precursor molecule in nitrogen fixation process, providing the necessary building blocks for the bacteria. This research proposes a metabolic cycle where plant-provided arginine and succinate fuel the nitrogen-fixing bacteria within the nodules.

The peak at 1426 cm^−1^, the last nitrogen-related peak, may result from overlapping peaks of various amino acids such as aspartic acid, tryptophan, valine, and methionine [33]. Nitrogen, in its structural molecule, contributes to the vibration at 1426 cm^−1^, where the bond is attributed to the carboxyl group connected to the amino group NH_2_. The accumulation of intensity from the peaks of these amino acids at this wavenumber can compensate for the low concentration of each individual amino acid in soybean plants. Although the combination of wavenumbers selected by our approach demonstrated effective performance in discerning biological nitrogen fixation (BNF), it is not inherently exclusive. The substantial collinearity among the spectral features suggests that alternative Raman wavenumbers could convey similar levels of information. Consequently, insights derived from these data might be transferable through different combinations of Raman shifts. As a result, models with diverse specifications and input variables may achieve comparable performance.

A key advantage of the method presented here is that it eliminates the need for sample preparation. Raman spectroscopy allows for the direct collection of spectra from live leaf samples, significantly reducing measurement costs. This contrasts favourably with existing methods like the ^15^N isotope dilution and natural abundance techniques. These methods require artificial enrichment of the soil with ^15^N-labelled fertiliser and the inclusion of reference crops near test plots, both of which can be cumbersome and expensive [42,43]. The Raman spectroscopy-based method explored in this research offers a more cost-effective and practical approach for BNF measurements.

Although the Raman method developed by Jochum et al. [13] provided excellent temporal resolution for tracking nitrogen fixation changes, its limitations include the need for a gas-collecting chamber enclosing the plant and potential interference from soil-released N_2_ due to denitrification. These factors hinder its practicality for high-throughput BNF measurement in field settings. Conversely, the optical spectroscopy method developed by Zhang et al. presented a low-cost option with high compatibility with IRMS-based quantification [44]. Our Raman-based method leverages the strengths of optical spectroscopy, such as low cost and rapid analysis, while offering additional advantages. Raman spectroscopy is insensitive to water interference and provides high molecular specificity, enabling us to develop models that require less calibration across diverse environmental conditions.

## 4. Conclusions

This study demonstrates that Raman spectroscopy can detect changes in the leaf spectra of legumes associated with biological nitrogen fixation (BNF) activity. Analysing these spectral changes allows for the estimation of nitrogen fixation levels in the plants.

The experiments demonstrated that using nitrogen abundance as a control parameter successfully created various levels of nitrogen fixation. However, this approach highlighted the importance of refining the nitrogen fixation model to account for the direct effects of nitrogen abundance on the model’s output. Additionally, the use of separate experimental groups for fixing and non-fixing plants proved to be an effective strategy for training and validating a model specifically designed to determine the BNF ratio in soybeans.

The partial least squares regression (PLSR) results for fixing plants supported our initial hypothesis. BNF, likely accompanied by metabolic changes, induced unique spectral signatures in the leaves. These signatures correlated well with the plants’ measured BNF rates. Notably, applying the model to non-fixing plants with varying nitrogen levels yielded minimal BNF values. This demonstrates that the model selectively identifies features indicative of BNF, rather than simply reflecting overall plant nitrogen content.

Since plant activity generates a diverse range of metabolites, information related to biological nitrogen fixation (BNF) might also be present in various chemical compounds and corresponding Raman bands. Consequently, the Raman bands identified in this study for BNF detection might not be entirely unique. However, the novelty lies in incorporating the structural characteristics of BNF-related molecules with multiple Raman peaks into a unified model derived from Raman spectroscopy data. Our findings confirm the presence of specific patterns in the Raman data that act as unique identifiers (fingerprints) of the BNF process. This allows us to leverage these patterns for quantifying nitrogen fixation. However, various factors influence BNF in legumes. Therefore, it is crucial to explore the impact of other parameters on the model’s performance to ensure its applicability across various field conditions.

## Data Availability

The original data presented in the study are openly available in FigShare at https://figshare.com/articles/dataset/dx_doi_org_10_6084_m9_figshare_25909780/25909780 (accessed on 28 May 2024).
